# Trends in the Incidence of Early-Onset Colorectal Adenocarcinoma Among Black and White US Residents Aged 40 to 49 Years, 2000-2017

**DOI:** 10.1001/jamanetworkopen.2021.30433

**Published:** 2021-11-09

**Authors:** Eric M. Montminy, Meijiao Zhou, Lauren Maniscalco, Harrison Penrose, Timothy Yen, Swati G. Patel, Xiao-Cheng Wu, Jordan J. Karlitz

**Affiliations:** 1 Tulane University School of Medicine, Division of Gastroenterology, New Orleans, Louisiana; 2 Louisiana State University Health Sciences Center, Department of Epidemiology; Louisiana Tumor Registry, New Orleans; 3 Tulane University School of Medicine, New Orleans, Louisiana; 4 University of Colorado School of Medicine, Division of Gastroenterology, Aurora; 5 Rocky Mountain Regional Veterans Affairs Medical Center, Aurora, Colorado; 6 Louisiana State University Health Sciences Center, Department of Public Health, New Orleans; 7 Denver Health Medical Center, University of Colorado School of Medicine, Denver

## Abstract

**Importance:**

Early-onset colorectal cancer incidence rates are rising faster in White individuals than Black individuals. However, prior National Cancer Institute Surveillance, Epidemiology, and End Results (SEER) racial stratification analyses used smaller SEER 13 databases, combined patients under age 50 years, did not stratify by sex, and did not focus on adenocarcinoma histologic subtypes (screening target).

**Objective:**

To perform a race- and sex-stratified adenocarcinoma incidence rate analysis in individuals aged 40 to 49 years using larger SEER 18 databases with expanded race data to better understand the colorectal cancer burden in those at or approaching screening age.

**Design, Setting, and Participants:**

This cross-sectional study used 2000 to 2017 SEER 18 annual age-adjusted colorectal cancer incidence rates stratified by anatomic subsite (colon or rectum), adenocarcinoma histology, race (non-Hispanic Black or non-Hispanic White), and sex for individuals aged 40 to 49 years, and yearly annual percent change (APC) incidence rates were calculated. Annual rate ratios (ARRs) between subgroups were determined. Statistical analysis was performed from January to March 2021.

**Main Outcomes and Measurements:**

Early-onset colorectal cancer incidence rates, APCs, and ARRs.

**Results:**

In this study, a total of 46 728 colorectal cancer cases were identified in 45 429 patients aged 40 to 49 years from 2000 to 2017. Among the 45 429 patients included in this study, 6480 (14.2%) were Black and 27 426 (60.4%) were White; the mean (SD) age was 45.5 (2.8) years. Among White individuals aged 40 to 49 years, colorectal adenocarcinoma incidence rates increased from 19.6 per 100 000 person-years in 2000 to 25.2 per 100 000 person-years in 2017 (APC, 1.6; 95% CI, 1.3 to 1.9). Among Black individuals aged 40 to 49 years, colorectal adenocarcinoma incidence rates were not significantly changed (26.4 per 100 000 person-years in 2000 and 25.8 per 100 000 person-years in 2017 [APC, –0.03; 95% CI, –0.5 to 0.5]). There were no significant differences in ARRs of absolute colorectal incidence rates between White and Black individuals from 2014 to 2017. Rectal-only absolute adenocarcinoma incidence rates in Black and White individuals remained similar from 2000 to 2008 but significantly diverged in 2009. As of 2017, rectal absolute incidence rates were 39% higher among White individuals than among Black individuals with increasing APC (APC, 2.2; 95% CI, 1.6 to 2.8) whereas rectal adenocarcinoma incidence rates among Black individuals were decreasing, although the APC was not statistically significant (APC, –1.4; 95% CI, –2.6 to 0.1). Absolute colonic adenocarcinoma incidence rates remained higher in Black individuals. The study subgroups with the largest divergence in APCs were rectal adenocarcinoma in White vs Black women (APC of 2.2 [95% CI, 1.6 to 2.8] vs APC of –1.7 [95% CI, –3.6 to 0.3], respectively).

**Conclusions and Relevance:**

This study found that colorectal adenocarcinoma incidence rates in people aged 40 to 49 years were increasing among White individuals but stabilized among Black individuals with absolute incidence rates becoming equivalent. Absolute rectal adenocarcinoma incidence rates were 39% lower in Black individuals with a widening disparity in rectal cancer between White and Black women. Possible contributors include introduction of a screening threshold of age 45 years in Black individuals in 2008. Although the average-risk screening age has now shifted to age 45 years in all racial groups, these data can help motivate real-world implementation of guidelines to maximize screening rates that have historically been suboptimal in younger individuals.

## Key Points

**Question:**

What are the trends in early-onset colorectal cancer incidence rates when people aged 40 to 49 years are assessed specifically and stratification by race, sex, and adenocarcinoma histology (the target of screening and prevention) is performed?

**Findings:**

In this cross-sectional study of 45 429 individuals aged 40 to 49 years with colorectal adenocarcinoma from 2000 to 2017, beginning in 2014, overall colorectal adenocarcinoma incidence rates were the same in Black and White populations, and rectal adenocarcinoma rates were 39.3% lower in Black individuals compared with White individuals with a widening disparity in rectal cancer between Black women and White women. Analyzing temporal changes in these rates suggest that these differences may have potentially arisen owing to historical availability of screening options for those aged at least 45 years among Black individuals but not White individuals.

**Meaning:**

These findings suggest that uniform implementation of screening options among individuals aged at least 45 years may help curtail rising colorectal cancer rates in other racial and ethnic groups; however, screening rates in younger patients are suboptimal and there will be need to motivate real-world implementation of guidelines.

## Teaser

This cross-sectional study examines trends in the incidence of early-onset colorectal cancer among Black and White US residents aged 40 to 49 years.

## Introduction

Incidence rates (IRs) are increasing for early-onset colorectal cancer (EOCRC), defined as CRC before age 50 years.^1^ This trend was identified using data from the National Cancer Institute Surveillance, Epidemiology, and End Result Program (SEER). More recent analyses found EOCRC absolute IRs are increasing faster in White individuals compared with Black individuals.^2^ These studies used SEER 13 databases (consisting of 13 cancer registries representing approximately 13% of the US population) and pooled all patients under age 50 years. SEER 13 has more limited race data compared with newer and larger SEER databases, including SEER 18 (18 cancer registries representing 28% of the US population).^3^ With expansion of race and ethnicity sampling, further clarification of racial disparities is necessary. Furthermore, with prior SEER studies pooling all patients under age 50 years and not performing a sex-stratified analysis, it is not possible to determine more specific race-stratified IR rate trends of people aged 40 to 49 years. Given recent controversy over initiating average-risk screening at age 45 years vs age 50 years, an understanding of the epidemiology of those at or approaching screening age is important.

In 2008, the American College of Gastroenterology (ACG) recommended that average-risk Black individuals initiate screening at age 45 years.^4^ In 2018, the American Cancer Society recommended average-risk screening begin in all patients at age 45 years. However, this recommendation was only formally adopted by the United States Preventive Services Task Force (USPSTF) in 2021.^5^ Even with the threshold finalized at age 45 years, screening rates in younger patients are markedly suboptimal (historically 28%-47% in individuals aged 50 to 54 years).^6^ Hence, a better understanding of the CRC burden in younger patients is required as similar issues with low screening rates, which can impede cancer prevention efforts, may be present when expanding screening to individuals aged 45 years. Race-stratified IR data of those in their 40s can also assess for changes in IRs between Black and White populations that may have arisen from differential average-risk screening recommendations since the 2008 ACG guideline.

In distinction to prior EOCRC studies, our study focuses on the adenocarcinoma histologic subtype specifically, the focus of screening, surveillance, and diagnostic testing. SEER databases pool all histologic subtypes, including carcinoids (neuroendocrine tumors), under the category colorectal cancer.^3^ Given recent evidence showing carcinoids are increasing faster than adenocarcinomas in young patients, which can affect IR reporting, an analysis of adenocarcinomas specifically is required.^7^

Hence, we used data from the 2000 to 2017 SEER 18 registries to assess colorectal, colon, and rectal adenocarcinoma IRs in individuals aged 40 to 49 years. We stratified by race (non-Hispanic Black and non-Hispanic White) and sex. In addition to assessing absolute IRs in subgroups and changes in these IRs over time via annual percent change (APC) analyses, we also calculated annual rate ratios (ARRs) to assess for significant differences of absolute IRs within subgroups.

## Methods

The Tulane University institutional review board considered this cross-sectional study exempt and granted waiver of informed consent because it used deidentified data. This study followed the Strengthening the Reporting of Observational Studies in Epidemiology (STROBE) reporting guideline.

### Study Design and Population

From January to March 2021, a cross-sectional analysis of the most recently published 2000 to 2017 SEER 18 annual age-adjusted CRC IRs of individuals aged 40 to 49 years was performed. Annual age-adjusted CRC IRs were stratified by anatomic subsite (colon vs rectum), adenocarcinoma histology, race (reported by participating SEER health care facilities, non-Hispanic Black and non-Hispanic White), and sex. Histologic and anatomic site coding is provided in the eAppendix in the Supplement.

### Statistical Analysis

Age-adjusted IRs were obtained from SEER*Stat software version 8.3.6 (National Cancer Institute), a public repository of case data and demographics reported from partnering cancer registry participants using strict SEER criteria.^8^ Joinpoint Regression Program version 4.8.0.1 (National Cancer Institute) quantified yearly APC trends in IRs per 100 000 person-years for subgroups (eAppendix in the Supplement for Joinpoint parameters). For APCs, a Monte Carlo permutation method was used in conjunction with provided standard errors from SEER*Stat to determine significance.^9^ Two-sided testing was used, and statistical significance was determined when *P* <  .05.

To determine statistically significant differences in absolute adenocarcinoma IRs within subgroups, ARRs were assessed. These were calculated for each calendar year and stratified by subgroups (race, sex, and anatomic subsite). Significant differences between racial subgroups were present when ARR 95% CIs did not cross 1 (see eAppendix in the Supplement). Statistical analysis was performed using Excel 365 software version (Microsoft) from January to March 2021.

## Results

In this study, a total of 46 728 CRC cases were identified in 45 429 patients aged 40 to 49 years from 2000 to 2017 (Table). Among the 45 429 patients included in this study, 6480 (14.2%) were Black and 27 426 (60.4%) were White; the mean (SD) age was 45.5 (2.8) years. In White individuals, 12 776 cases were identified in women and 15 443 in men. In Black individuals, 3282 cases were identified in women and 3371 in men.

**Table.** SEER 18 Colorectal Adenocarcinoma Demographic Characteristics for Ages 40 to 49 Years

Characteristics	Patient counts, No. (n=45 429)		Case counts, No. (n = 46 728)	
	Non-Hispanic Black	Non-Hispanic White	Non-Hispanic Black	Non-Hispanic White
Colorectal				
Women	3199	12 496	3282	12 776
Men	3281	14 930	3371	15 443
Colon-only				
Women	2469	7641	2535	7829
Men	2264	8491	2316	8822
Rectal-only				
Women	730	4855	747	4947
Men	1017	6439	1055	6621

### Combined Sex Black and White Colorectal Adenocarcinoma IR Comparisons

**Figure 1.**
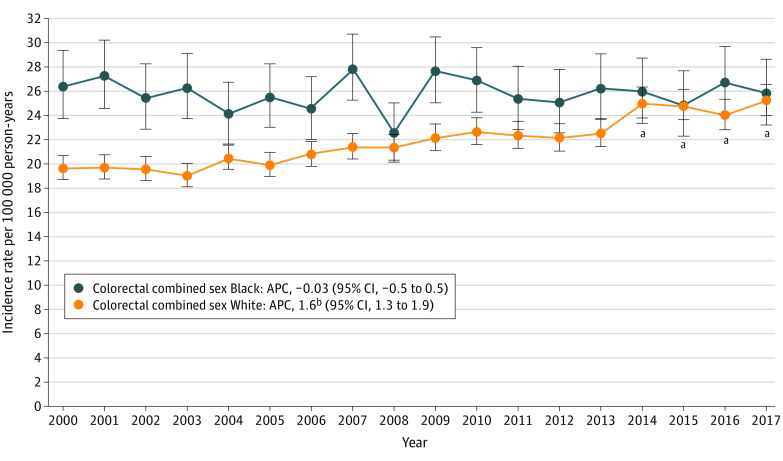
National Cancer Institute Surveillance, Epidemiology, and End Results 18 Colorectal Adenocarcinoma Incidence Rates in Black US Residents vs White US Residents Aged 40 to 49 Years, 2000-2017 APC indicates annual percent change. ^a^Annual incidence rates of colorectal adenocarcinoma among White individuals were not significantly different than among Black individuals from 2014 to 2017. ^b^APC significantly changing with *P* < .05.

Among White individuals, colorectal adenocarcinoma IRs significantly increased from 19.6 per 100 000 person-years in 2000 to 25.2 per 100 000 person-years in 2017 (APC, 1.6; 95% CI, 1.3-1.9) (Figure 1). Among Black individuals, colorectal adenocarcinoma IRs did not significantly change (26.4 per 100 000 person-years in 2000 and 25.8 to 100 000 person-years in 2017; APC, –0.03 95% CI, –0.5 to 0.5). After 2013, colorectal adenocarcinoma IRs among Black individuals were no longer significantly higher than among White individuals (no significant difference in ARRs).

### Combined Sex Black and White Adenocarcinoma IR Comparisons by Subsite

**Figure 2.**
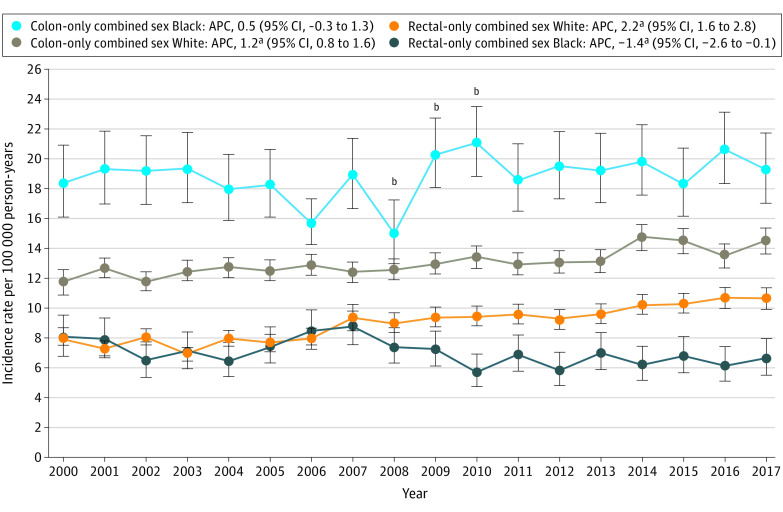
National Cancer Institute Surveillance, Epidemiology, and End Results 18 Colon-Only and Rectal-Only Adenocarcinoma Incidence Rates in Black US Residents and White US Residents Aged 40 to 49 Years, 2000-2017 From 2000 to 2008, annual incidence rates of rectal adenocarcinoma among White individuals were not significantly different than among Black individuals. From 2009 to 2017, annual incidence rates of rectal adenocarcinoma among White individuals were significantly higher than among Black individuals. APC indicates annual percent change. ^a^APC significantly changing with *P* < .05. ^b^There was a 41.1% increase in colon cancer incidence rates in Black individuals from 2008 to 2010.

Within the colon subsite, absolute IRs remained significantly higher among Black individuals than White individuals during the entire 2000 to 2017 period (Figure 2). However, from 2000 to 2017, colon adenocarcinoma IRs did not significantly change among Black individuals (APC, 0.5; 95% CI, –0.3 to 1.3) and colon adenocarcinoma IRs significantly increased among White individuals (APC, 1.2; 95% CI, 0.8-1.6). Regarding rectal subsites, absolute rectal adenocarcinoma IRs prior to 2009 among Black individuals were not significantly different than White individuals (Figure 2). However, starting in 2009, rectal adenocarcinoma IRs significantly diverged. As of 2017, rectal absolute IRs among White individuals were 39.3% higher than in Black individuals with an increasing APC of 2.2 (95% CI, 1.6 to 2.8), whereas rectal adenocarcinoma IRs among Black individuals were significantly decreasing (APC, –1.4; 95% CI, –2.6 to –0.1).

### Colorectal Adenocarcinoma IR Comparisons by Sex and Race

**Figure 3.**
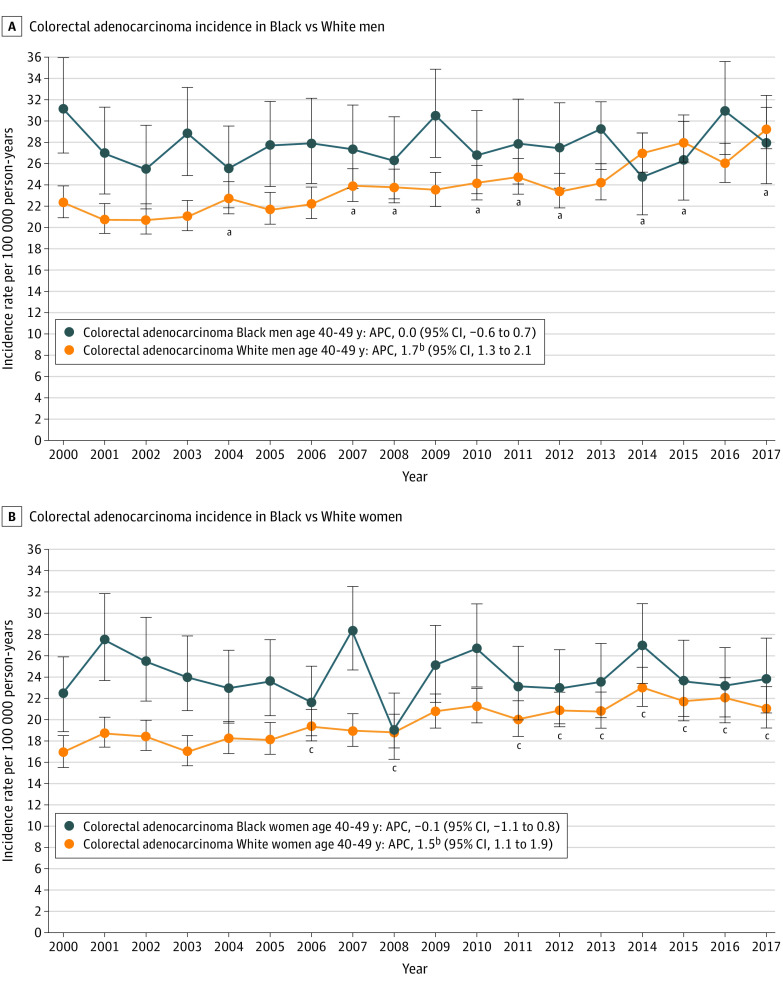
National Cancer Institute Surveillance, Epidemiology, and End Results 18 Colorectal Adenocarcinoma Incidence Rates in Black US Residents and White US Residents Aged 40 to 49 Years, Stratified by Sex, 2000-2017 APC indicates annual percent change. ^a^Annual incidence rates of colorectal adenocarcinoma among White men were not significantly different than among Black men in 2004, 2007, 2008, 2010, 2011, 2012, 2014, 2015, and 2017. ^b^APC significantly changing with *P* < .05. ^c^Annual incidence rates of colorectal adenocarcinoma among White women were not significantly different than among Black women in 2006, 2008, and 2011-2017.

Colorectal adenocarcinoma IRs significantly increased among White men from 2000 to 2017 (APC, 1.7; 95% CI, 1.3 to 2.1) whereas IRs did not significantly change among Black men (APC, 0.0; 95% CI, –0.6 to 0.7) (Figure 3). Starting in 2007, based on ARR comparisons, absolute colorectal adenocarcinoma IRs were the same in Black men and White men (except in 2009, 2013, and 2016).

Colorectal adenocarcinoma IRs significantly increased among White women from 2000 to 2017 (APC, 1.5; 95% CI, 1.1 to 1.9), whereas colorectal adenocarcinoma IRs remained unchanged among Black women (APC, −0.1; 95% CI, –1.1 to 0.8) (Figure 3). After 2010, yearly absolute colorectal adenocarcinoma IRs among Black women were not significantly different than White women.

### Colorectal Adenocarcinoma IR Comparisons by Sex, Race, and Subsite (Colon and Rectum)

**Figure 4.**
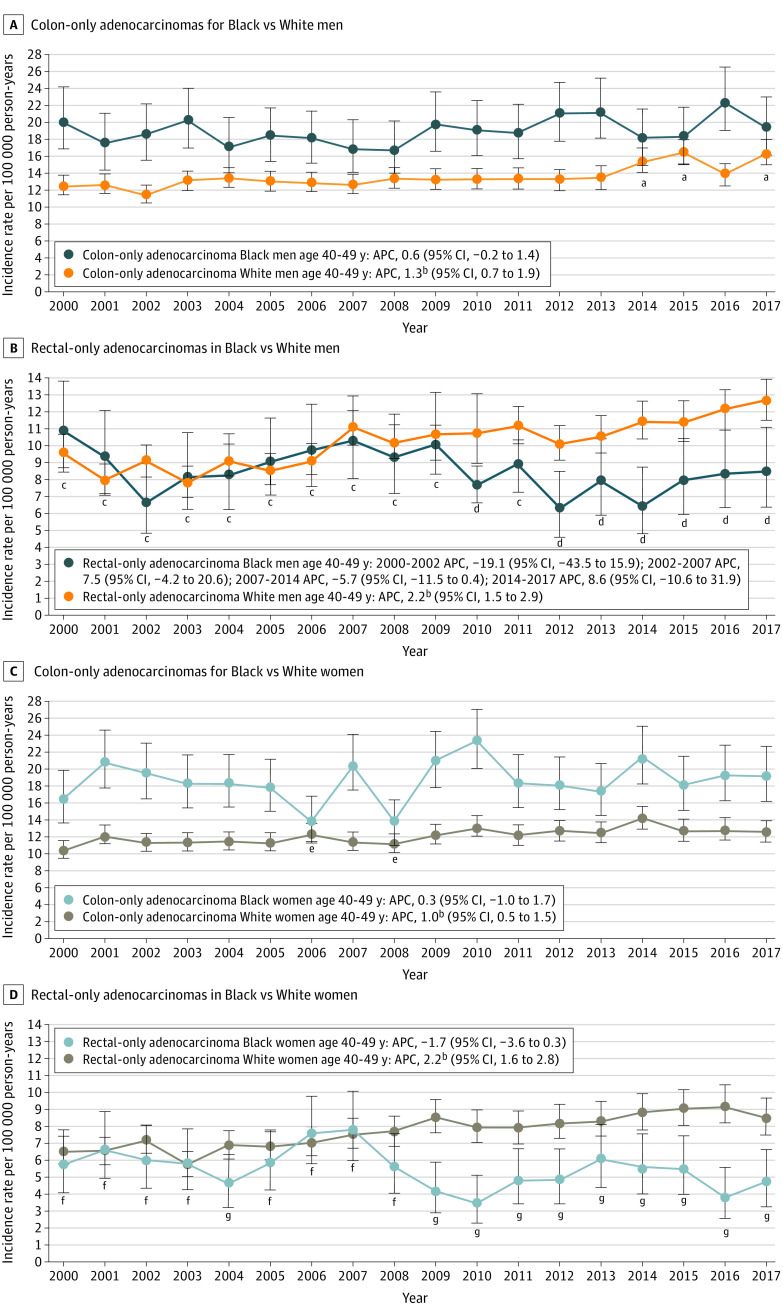
National Cancer Institute Surveillance, Epidemiology, and End Results 18 Colon-Only and Rectal-Only Adenocarcinoma Incidence Rates in Black US Residents and White US Residents Aged 40 to 49 Years, Stratified by Sex, 2000-2017 APC indicates annual percent change. ^a^Annual incidence rate of colon-only adenocarcinoma among Black men was not significantly different than among White men in 2014, 2015, and 2017. B, Annual incidence rate of rectal-only adenocarcinoma among Black men was not significantly different than among White men from 2000 to 2009 and in 2011. C, Annual incidence rate of colon-only adenocarcinoma among Black women was not significantly different than among White women in 2006 and 2008. D, Annual incidence rate of rectal-only adenocarcinoma among Black women was not significantly different than among White women in 2000 to 2003 and 2005 to 2008. Annual incidence rate of rectal-only adenocarcinoma was significantly higher among White women than Black women in 2004 and 2009 to 2017. ^b^APC significantly changing with *P* < .05. ^c^Annual incidence rates of rectal-only adenocarcinoma among Black men were not significantly different than among White men from 2000 to 2009 and in 2011. ^d^ Annual incidence rates of rectal-only adenocarcinoma were significantly higher among White men than Black men in 2010 and 2012 to 2017. ^e^Annual incidence rates of colon-only adenocarcinoma among Black women were not significantly different than among White women in 2006 and 2008. ^f^Annual incidence rates of rectal-only adenocarcinoma among Black women were not significantly different than among White women in 2000 to 2003 and 2005 to 2008. ^g^Annual incidence rates of rectal-only adenocarcinoma were significantly higher among White women than Black women in 2004 and 2009 to 2017.

Colon adenocarcinoma IRs have been increasing among White men (APC, 1.3; 95% CI, 0.7 to 1.9) and women (APC, 1.0; 95% CI, 0.5 to 1.5) whereas they have been unchanged among Black men (APC, 0.6; 95% CI, −0.2 to 1.4) and women (APC, 0.3; 95% CI, −1.0 to 1.7) (Figure 4). Yearly absolute colon adenocarcinoma IRs remained higher among Black men than those of White men until 2014 when there was no significant difference between absolute IRs (except in 2016). Absolute colon adenocarcinoma IRs in Black women remained higher than in White women in all years except 2006 and 2008.

Rectal adenocarcinoma IRs significantly increased among White men and White women from 2000 to 2017 at the same APCs (2.2; 95% CI, 1.6-2.8). In comparison, APCs among Black men and Black women did not significantly change. The study subgroups with the largest divergence in APCs were rectal adenocarcinoma in White vs Black women (APC of 2.2 [95% CI, 1.6 to 2.8] vs APC of −1.7 [95% CI, −3.6 to 0.3], respectively). Rectal adenocarcinoma absolute IRs were significantly higher among White men than Black men each year after 2011. Rectal adenocarcinoma IRs were significantly increased among White women compared with Black women each year after 2008 (Figure 4).

### Temporal Association of IR Changes to 2008 ACG Guidelines to Screen African Americans at Age 45 Years

Coincident with the 2008 ACG guidelines, as can be seen in Figure 2, there was a 41.1% increase in colon cancer rates from 2008 to 2010 in Black individuals (15.1 per 100 000 person-years in 2008 to 21.3 per 100 000 person-years in 2010). Based on incidence rate ratio analysis, this increase reached statistical significance during 2008 to 2009 (eFigure in the Supplement).

## Discussion

We provided a race- and sex-stratified IR analysis in individuals aged 40 to 49 years. By using SEER 18, with expanded race data compared with SEER 13 and focusing on adenocarcinomas specifically, to our knowledge we reported the most comprehensive assessment of EOCRC IRs in this age group. Individuals aged 40 to 49 years are important to analyze given recent controversy regarding average-risk screening at age 45 years vs age 50 years and the need to understand CRC burden in those at or approaching screening age. Even though the age 45 years–threshold has been formally approved by the USPSTF, CRC screening rates in younger patients are suboptimal (historically 28%-47% in individuals aged 50 to 54 years) and an understanding of IRs and risk can help motivate real-world implementation of guidelines to maximize screening rates during expansion to younger individuals.^5^

Key findings include that absolute colorectal adenocarcinoma rates are now equivalent in White and Black individuals. Furthermore, although absolute colon adenocarcinoma IRs in Black individuals are higher than in White individuals, colon adenocarcinoma APCs in Black individuals have remained stable in comparison with White individuals in whom IRs are increasing. Rectal adenocarcinomas have been significantly decreasing in Black individuals and absolute rectal adenocarcinoma rates are now 39.3% lower in Black individuals compared with White individuals. This finding contrasts with a prior analysis in which rectal cancer rates were found to be increasing in both White and Black individuals, although at a less steep pace in Black individuals.^2^ Sex stratification has revealed that in recent years, absolute colon adenocarcinoma IRs among Black men are similar to rates among White men, although absolute colon adenocarcinoma IRs in Black women have remained consistently higher than in White women. The subgroup with the largest divergence in APCs is rectal cancer in White women (APC, 2.2) vs rectal cancer in Black women (APC, –1.7). Furthermore, rectal adenocarcinoma APCs are the same in White women and White men. This is of particular interest given that, historically, the largest sex disparity (IRs greater in men than women) for CRC has been for rectal subsites (male-to-female incidence rate ratio [IRR], 1.62; 95% CI, 1.60-1.63).^10^

Focusing on race, a possible contribution to our findings is a 2005 paper recommending screening for African Americans at age 45 years and the formal 2008 ACG guideline consolidating this threshold.^4,11^ Rectal adenocarcinomas in Black individuals became significantly lower compared with White individuals starting in 2009. There was also a steep 41.1% increase in colon cancer rates from 2008 to 2010 in Black individuals (15.1 per 100 000 person-years in 2008 to 21.3 per 100 000 person-years in 2010), which reached statistical significance over 2008 to 2009. This finding could be consistent with initial detection of preclinical colonic lesions owing to screening initiation. Stabilization in colon adenocarcinoma rates and decreases in rectal adenocarcinoma rates thereafter would be ultimately consistent with cancer prevention. The fact that a corresponding steep colon adenocarcinoma rate increase was not seen in White individuals from 2008 to 2010, in whom screening at age 45 years was not recommended, lends support to this assessment. Furthermore, a recent 2020 publication found a steep 46.1% increase in CRC IRs from the age of 49 years to 50 years (when average risk screening has traditionally commenced), suggesting that numerous preclinical cancers can be revealed when a population is subject to screening initiation.^12^ Overall, our findings provide indirect support that earlier screening at age 45 years could also beneficially affect rising CRC IRs in White individuals. Importantly, a recent staging analysis of individuals aged 40 to 49 years found that more advanced stage disease is being discovered in this age group.^13^ This lends support to the thought that the continued increase in CRC IRs in White individuals in our analysis is because of a true increase in CRC burden as opposed to increased detection from screening utilization (which would be expected to detect earlier stage disease).^13^

Recently, Vajravelu et al^14^ performed a retrospective cohort study using US medical claims-based data of 88 million enrollees of large commercial and Medicare Advantage health plans. In this analysis, 9% of Black individuals aged 45 years were evaluated for CRC screening (using lower endoscopy or stool-occult testing) by age 46 years. Although a modest increase, these data demonstrate a clear screening initiation effect and support our findings of a shift in IRs in the Black population in the years surrounding this ACG recommendation. The change in screening was only assessed within 1 age year of 45 (screening by age 46 years) in the study by Vajravelu et al.^14^ Hence, there may be underestimation of a screening increase as some individuals may initiate screening more than 1 year after the age recommendation (ie, start screening at age 47 years, 48 years, or later.).

To our knowledge, our analysis is the first to find that White women may be a significant driver of overall increases in rectal cancer rates. Although obesity may be a significant contributor to rising EOCRC rates in general, obesity rate increases have not been found to be disproportionately higher in White women, and thus other underlying etiologies may be responsible for the increasing IRs of rectal cancer.^15,16^ In terms of other modifiable CRC risk factors, the Centers for Disease Control has demonstrated that physical inactivity, tobacco, and heavy alcohol use are not increasing disproportionately in women compared with men under age 50 years in the past 2 decades.^17^ For tobacco and alcohol use, abuse prevalence is higher in men under age 50 years compared with women.^17^ This suggests that such risk factors may not be driving the disproportionate increases in EOCRC in White women and that further studies with larger racial cohorts will be required. It is possible that differential CRC screening exposure may help to explain the difference in rectal cancer rates between Black women and White women, with Black women historically having available screening options at age 45 years for prevention compared with White women.

An important distinction between our study and prior studies is the analysis of colorectal adenocarcinomas. Adenocarcinomas are the target of screening, surveillance, and diagnostic testing, and thus it is important to understand the epidemiology of this histologic subtype. A recent study found that in young patients with CRC, carcinoids (neuroendocrine tumors which are classified as colorectal cancer by SEER registries) have been outpacing adenocarcinoma rates in recent years.^7^ These changes were driven by rectal subsites and were most pronounced in persons aged 50 to 54 years, in whom rectal carcinoid tumors increased by 159% (2.36 to 6.10 per 100 000 person-years) between 2000 to 2002 and 2014 to 2016, compared with 10% for adenocarcinoma (18.07 to 19.84 per 100 000 person-years), ultimately accounting for 22.6% of all rectal cancer cases.^7^ Among individuals age 40 to 49 years, colorectal carcinoids increased by 38% between 2000 and 2016, colon carcinoids increased by 18%, and rectal carcinoids increased by 42%.^7^ These findings underscore the importance of stratifying EOCRC rates by histology as performed in our analysis.

A recent study by Chang et al^18^ used the large United States Cancer Statistics database, which combines both SEER and National Program of Cancer Registries (NPCR) data, to analyze EOCRC rates in various young subgroups, including individuals aged 40 to 49 years.^18^ They found that White individuals were the only racial group with a consistent CRC increase in incidence across all younger ages. However, analysis of histologic subtypes (ie, adenocarcinomas) was not reported. Other key differences between our analysis and theirs include our group analyzing temporal trends and APC shifts to assess potential screening effects (including introduction of a screening recommendation in 2008 for Black individuals aged 45 years) and reporting subsite data (rectal vs colon cancer) stratified by both race and sex for individuals aged 40 to 49 years specifically.

Our study provides additional context to public health policy. Our findings that IRs in Black individuals are unchanged or decreasing compared with White individuals helps lend support that screening initiation in younger patients of all races has the potential to curb rising CRC rates. However, although cancer IRs are important measurements to understand implications of earlier screening, analyzing CRC case counts can provide additional perspective. In a previous study, our group determined that from 2000 to 2015, there were approximately 129 000 cases of CRC nationally (43 000 during a 5-year period) in patients aged 45 to 50 years.^12^ Many of these cancers could be diagnosed at an earlier stage or prevented altogether with screening at age 45 years instead of age 50 years. These case counts were higher than those in a modeling study which estimated that 29 400 CRC cases over 5 years could be prevented with screening at age 45 years. In this modeling study, the cost to implement screening at age 45 years was thought to be likely cost-effective.^19^

### Limitations and Strengths

This study had some limitations and strengths. Limitations include the use of population-based observational data. Thus, they do not provide the mechanisms leading to the changes in EOCRC rates and prevent assessing other factors that can shift IRs, including screening access and health care clinician recommendations for screening. Furthermore, due to sample size limitations our analysis focused on IRs in non-Hispanic Black and non-Hispanic White patients and did not include other groups including Asian individuals, Hispanic individuals, and others.

Strengths include the use of SEER 18 data, which includes more expansive race data compared with SEER 13, which had been used in a prior analysis. In addition, our focus on individuals aged 40 to 49 years and the adenocarcinoma histologic subtype provided a comprehensive understanding of EOCRC in this age group, which is necessary given recent controversies regarding average-risk CRC screening recommendations and concerns about suboptimal screening rates in younger patients.

## Conclusions

This cross-sectional study found that CRC rates among those aged 40 to 49 years are continuing to increase in White individuals but have remained unchanged in Black individuals. Absolute colorectal adenocarcinoma IRs are now the same in both Black and White populations. Rectal adenocarcinoma rates have been steadily decreasing in Black individuals and are now 39.3% lower than in White individuals. Absolute colon adenocarcinoma rates in Black men are now similar to White men. As revealed by the timing of these IR differences, a potential driver may be earlier adoption of average-screening in Black individuals, which would suggest that earlier screening at age 45 years could also benefit other populations. The subgroups with the greatest disparity in rates of change are rectal cancer in White women compared with Black women. In contrast to Black women, rectal adenocarcinoma rates are steadily increasing in White women. The cause of this disparity is unknown but could also be due to differences in CRC screening exposure. However, further study will be required of these populations and could reveal differential risk factors that could also help us better understand the rising EOCRC rates we have been seeing in general. Despite increasing colorectal adenocarcinoma burden in White individuals compared with Black individuals aged 40 to 49 years, there remains a higher burden of colorectal cancer in older Black individuals, which requires ongoing attention.^18^ Furthermore, absolute colon-only adenocarcinoma in 40 to 49 year-old Black women remained higher than White women and further research is needed in women to clarify this difference.

The high-risk groups we have identified underscore the importance of optimizing colorectal cancer screening implementation. Even with recent formal approval of the screening age threshold of 45 years, prior analyses have demonstrated that screening rates in younger patients are suboptimal. Thus, the CRC risk we have demonstrated in our analyses can help motivate real-world implementation of screening recommendations.

## Notes

Supplement. **eAppendix.** Supplemental Materials **eFigure.** Annual Incidence Rate Ratios of Age 40-49 Black American Colon-only Adenocarcinoma Over 2000-2017
