# Error in Results Section of Abstract

**DOI:** 10.1001/jamanetworkopen.2021.43726

**Published:** 2021-12-17

In the Original Investigation titled “Trends in the Incidence of Early-Onset Colorectal Adenocarcinoma Among Black and White US Residents Aged 40 to 49 Years, 2000-2017,”^1^ published November 9, 2021, there was an error in the data of the Results section. The rectal adenocarcinoma incidence rates among Black individuals were significantly decreasing, with an annual percent change of –1.4 (95% CI, –2.6 to –0.1). This article has been corrected.^1^
