# Supplementary material for: Trends in the Incidence of Early-Onset Colorectal Adenocarcinoma Among Black and White US Residents Aged 40 to 49 Years, 2000-2017
Source: JAMA Netw Open. 2021 Nov 9;4(11):e2130433. doi: 10.1001/jamanetworkopen.2021.30433 (PMC8579235; doi:10.1001/jamanetworkopen.2021.30433)
Supplement: Supplement. — eAppendix. Supplemental Materials eFigure. Annual Incidence Rate Ratios of Age 40-49 Black American Colon-only Adenocarcinoma Over 2000-2017 [file jamanetwopen-e2130433-s001.pdf]

Supplemental Online Content

Montminy EM, Zhou M, Maniscalco L, et al. Trends in the incidence of early-onset colorectal adenocarcinoma among Black and White US residents aged 40 to 49 years, 2000-2017. *JAMA Netw Open.* 2021;4(10):e2130433. doi:10.1001/jamanetworkopen.2021.30433

eAppendix. Supplemental Materials

eFigure. Annual Incidence Rate Ratios of Age 40-49 Black American Colon-only Adenocarcinoma Over 2000-2017

This supplemental material has been provided by the authors to give readers additional information about their work.

This supplemental material has been provided by the authors to give readers additional information about their work.

© 2021 Montminy EM et al. *JAMA Network Open.*

eAppendix. Supplemental Methods

Institutional Review Board Disclosure

The study was reviewed by the Tulane University Biomedical Institutional Review Board and considered exempt given its use of deidentified data.

SEER 18 Registries

SEER 18 database consists of 18 specific regional registries: Alaska Native Tumor Registry, Connecticut, Detroit, Georgia Center for Cancer Statistics (Atlanta, Greater Georgia, Rural Georgia), Greater Bay Area Cancer Registry (San Francisco-Oakland and San Jose-Monterey), Greater California, Hawaii, Iowa, Kentucky, Los Angeles, Louisiana, New Mexico, New Jersey, Seattle-Puget Sound, and Utah.

Joinpoint Parameters

Joinpoint Version 4.8.0.1 was utilized. Independent variable was selected as "Year". Age-adjusted rates and Standard Errors were provided from the 2018 SEER 18 submission with in situ cases and excluded appendix cases. Heteroscedastic Error Option was set to default standard error (provided). Log Transformation was set to default "Yes {ln(y) = xb}. Maximum number of joinpoints selected was no more than 4. Default minimal number of observations from a joinpoint to either end of the data was 2. Default minimal number of observations between two joinpoints was 2. Default number of points to place between adjacent observed x values in the grid search was 0. Permutation Test: overall significance level was 0.05 and number of permutations was 4499. Autocorrelated Errors Option was selected to default "Fit an uncorrelated errors model". APC Confidence Interval was set to default parametric Method. AAPC Segment Ranges was set to entire range. Advanced analysis tool was set to no advanced analyses.

© 2021 Montminy EM et al. *JAMA Network Open.*

Histologic/Anatomic Site Coding

The Recode ICD-O-3 site/histology codes and behavior codes were used to define adenocarcinomas. The in situ cases (behavior code=2) and invasive/malignant cases (behavior code=3) only were included. To make the eligibility criteria clearer, we included the ICD-O-3 topographic (anatomic site) codes [C180-C189, C199, C209, and C260, excluding C181 (appendix)] that we used to define colorectal cancer case sites as defined in Fritz A, Percy C, Jack A, *et al*. Eds., International Classification of Diseases for Oncology, 3rd ed., Geneva: World Health Organization, 2000.

The colorectal, colon-only, and rectal-only cancers were defined by the ICD-O-3 codes C180-C189, C199, C209, and C260. Adenocarcinoma coding 447 included 8140/3, 8141/3, 8143/3, 8144/3, 8210/3, 8211/3, 8213/3, 8220/3, 8221/3, 8260-8265/3, 448 8255/3, 8260-8263/3, 8310/3, 8323/3, 8440/3, 8460/3, 8470/3, 8472/3, 8480-8482/3, 8570/3, 449 8574/3, 8576/3. In situ cases were included in our study.

© 2021 Montminy EM et al. *JAMA Network Open.*

eFigure. Annual Incidence Rate Ratios of Age 40-49 Black American Colon-only Adenocarcinoma over 2000-2017

Supplemental Figure 1

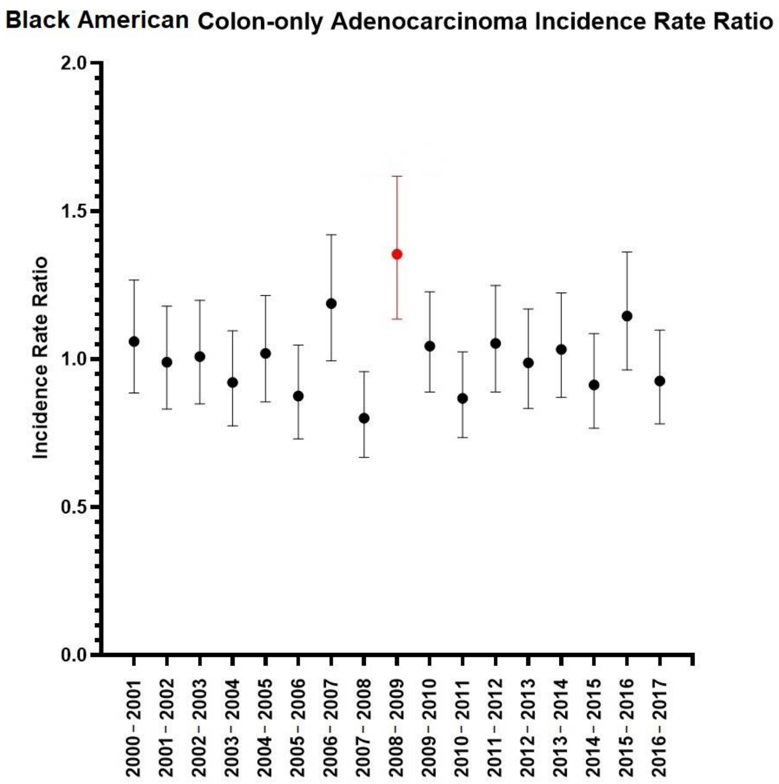

Supplemental Figure 1. Year-to-year incidence rate ratios with 95% confidence intervals are depicted. The red point indicates the incidence rate in 2009 was significantly higher than the incidence rate in 2008 (incidence rate ratio did not cross one, $p<0.05$).

© 2021 Montminy EM et al. *JAMA Network Open.*
